# Late gadolinium enhancement is not related to the severity of aortic regurgitation. A single center study

**DOI:** 10.1186/1532-429X-18-S1-Q55

**Published:** 2016-01-27

**Authors:** Henrique S Trad, Ana Marta A Gali, Marcel Koenigkam Santos, Maria Fernanda Braggion-Santos, Gustavo J Volpe, Benedito C Maciel, Andre Schmidt

**Affiliations:** 1 Cardiology, HCFMRP, Ribeirao Preto, Brazil; 2 Radiology, CCIFM, HCFMRP, Ribeirao Preto, Brazil

## Background

Myocardial fibrosis as detected by late gadolinium enhancement (LGE) has been linked to adverse prognosis in several cardiovascular diseases. In aortic stenosis, LGE has been linked to worse systolic function and prognosis. In aortic regurgitation (AR), LGE has only been described in small experimental and clinical trials.

Objectives: To evaluate the presence of LGE, its relation to left ventricular function and volumes, and the severity of AR in a cohort of patients.

## Methods

98 consecutive patients with isolated AR underwent cardiac magnetic resonance (CMR) with specific sequences in order to evaluate cardiac size and function, aortic regurgitant volume and LGE. Descriptive statistical analysis and Fisher's exam test were performed as appropriate to identify the relations between presence of LGE and AR severity.

## Results

98 patients with chronic isolated AR were selected. Aortic regurgitation severity as defined by regurgitant volume was as follows: 44 patients (45%) mild, 34 (35%) moderate and 20 (20%) severe. LGE was present in 16 patients (16.3%). Only patient age was significantly higher in patients with LGE (64.3 ± 10.5 vs.52.6 ± 17.4, p = 0.01) compared to the non-LGE group. In relation to morphologic and functional parameters (ejection fraction, end systolic and end diastolic volumes, and AR severity), there was no statistical difference between patients with or without LGE.

## Conclusions

Myocardial fibrosis as detected by LGE is not uncommon in patients with AR, although it is apparently related to aging and not to the severity o AR or its functional consequences.

